# Natural Products and Disease Prevention, Relief and Treatment

**DOI:** 10.3390/nu14122396

**Published:** 2022-06-09

**Authors:** Md Soriful Islam

**Affiliations:** Department of Gynecology and Obstetrics, Johns Hopkins University School of Medicine, 720 Rutland Ave, Ross Research Building, Room 624, Baltimore, MD 21205, USA; soriful84@gmail.com; Tel.: +1-410-614-2000; Fax: +1-410-614-7060

**Keywords:** Rubi Fructus, Mokko lactone, YG-1 extract, *Dracocephalum moldavica* ethanol extract, lactoferrin, *Sparassis crispa*, fisetin and quercetin, *Sargassum plagiophyllum* extract, inflammation, fibrosis

This Special Issue focusses on the role of natural products in disease prevention, relief and treatment. Natural products are known to regulate key pathophysiological processes, such as inflammation, fibrosis, hypoxia, oxidative stress, cell proliferation, angiogenesis, migration, and metabolism, that are linked to human diseases. In this Special Issue, we have published a number of high-quality manuscripts that bring attention to the emerging role of natural products in a wide range of human diseases, including inflammatory and fibrotic diseases, as well as cancers. This editorial will discuss individual reports published in this Special Issue.

Rubi Fructus, the unripe fruits of *Rubus coreanus* Miquel., is well known for its different active biological properties. Kim et al. examined the effect of Rubi Fructus (RF) on inflammatory signaling pathways in RAW 264.7 macrophages [1]. The data showed that RF was effective as an anti-inflammatory product. RF exhibited anti-inflammatory activity on LPS-stimulated macrophages by regulating a series of inflammatory mediators, including IL6 (interleukin 6), MCP-1 (monocyte chemotactic activating factor 1), TNF-α (tumor necrosis factor-α), as well as their associated signaling pathways (such as STAT, JAK2, c-Jun, and CHOP) [1].

Mokko lactone (ML), a naturally occurring guaianolide sesquiterpene, is known for its antioxidant and anti-inflammatory properties. Sirwi et al. investigated the protective effect of ML in doxorubicin (DOX)-induced cardiotoxicity [2]. Rats were treated with ML for 10 days, followed by one IP injection of DOX. The results showed a significant protective effect of ML in DOX-induced cardiotoxicity in terms of amelioration of oxidative stress, apoptosis, and inflammation [2]. These findings suggest that ML can be used to alleviate DOX-induced cardiotoxicity in patients.

YG-1 extract is a mixture of three different plants, including Lonicera japonica, Arctii Fructus, and Scutel lariae radix. Kim and coworkers investigated the effect of different concentrations of YG-1 extract on bronchodilatation, as well as acute bronchial and pulmonary inflammation relief ex vivo and in vivo, respectively [3]. Ex vivo, YG-1 extract showed concentration-dependent bronchodilation effects in Sprague Dawley rats by upregulating cAMP (cyclic adenosine monophosphate) levels through the β2-AR (β2-adrenergic receptor)/PKA pathway [3]. In vivo, the effects of YG-1 extract on acute bronchial and pulmonary inflammation were investigated in C57BL/6 mice. The results showed that YG-1 extract treatment reduced the prevalence of respiratory symptoms and the incidence of non-specific lung diseases. YG-1 treatment also improved acute bronchial and pulmonary inflammation, at least in part, by regulating the inflammasome signaling pathways (such as NLRP3/caspase-1) [3]. These results support further exploration of YG-1 extract as an effective natural product in developing therapeutics for respiratory diseases.

DMEE is an ethanol extract from the herb *Dracocephalum moldavica*, which contains the active compound oleanolic acid. Kim et al. demonstrated the anti-inflammatory effect of DMEE on LPS-induced inflammatory responses in 264.7 macrophages and murine model of sepsis. The data showed that that DMEE effectively reduced LPS-induced inflammatory responses in RAW 264.7 macrophages and LPS-induced septic shock mice. These effects were mediated, at least in part, by inhibiting MAPK and NF-κB signaling pathways (such as JNK, ERK and p65), as well as reducing the production of inflammatory cytokine IL6. These findings are interesting and significant for future research in the area of natural products for sepsis [4].

Lactoferrin (LF) is an iron-binding glycoprotein found in all exocrine fluids, including tears, sweat, and saliva. It is also abundant in milk. Aoyama et al. investigated the possible antioxidant, anti-inflammatory and antitumoral activities of lactoferrin in a combined high-fat diet/dimethylnitrosamine treated C×32 dominant negative transgenic (C×32ΔTg) rat model of NASH (non-alcoholic steatohepatitis). Lactoferrin showed a selective chemopreventive effect against liver tissue injury and progression via regulating the NF-κB /TGF-β1 signaling pathways [5].

Sparassis (also known as cauliflower mushroom), a genus of parasitic and saprobic mushrooms, is characterized by its unique shape and appearance. Nowacka-Jechalkee et al. investigated the chemical composition and potential biologic activity of crude polysaccharides isolated from *Sparassis crispa* (CPS). CPS mainly consists of carbohydrates and exerts cytotoxic effects on colon cancer cells. Interestingly, CPS was found to be non-toxic to normal human colon epithelial cells. CPS showed moderate antioxidant activity and inhibited inflammatory molecules, such as COX-2 [6]. These results suggest that CPS may be important as a part of the regular human diet. Further study is needed to explore its potentiality as a chemopreventive and therapeutic agent in colon cancers.

Circulating uric acid is thought to be effective against loss of bone mineral density and oxidative damage. However, excess serum uric acid is associated with diseases such as gout, hypertension, and cardiovascular disease. URAT1, a member of the OAT (organic anion transporter) family, is an anion-exchanging uptake transporter localized to the apical membrane of renal proximal tubular cells. URAT1 regulates most urate reabsorption into blood; therefore, the inhibition of URAT1 may help decrease serum urate levels by increasing the net renal urate excretion. Toyoda et al. brings to attention the beneficial effect of natural compounds on the inhibition of URAT1. This group used cell-based urate transport assay to investigate the inhibitory effects of 162 extracts of plant materials on URAT1. They found that fisetin and quercetin showed significant inhibitory effects on URAT1 cell-based urate transport assay [7]. Overall, this study pointed out that some selective phytochemicals should be investigated further in human studies, and may provide new clues for using nutraceuticals to promote human health.

Seaweed is widely known for its beneficial role for human health. Khuituan et al. examined the effects of *Sargassum plagiophyllum* extract (SPE) on constipated mice, particularly the functions of the gastrointestinal tract and gut microbiota [8]. SPE contains phenolic compounds and carotenoids (such as fucoxanthin), as well as long-chain-sulfated polysaccharides (such as fucoidan). This study showed that SPE pretreatment increased the frequency of gut contraction, leading to reduced gut transit time. The beneficial effect of SPE may be due to the presence of bioactive compounds. Overall, SPE might be useful for the development of human food supplements to prevent constipation.

Subramaiam et al. examined the immunomodulatory, anti-metastatic, gene expression analysis and anticancer effects of combined Spirulina and γT3 against breast cancer using a syngeneic mouse model [9]. They found that the combination of Spirulina and γT3 does not appear to have any synergistic anticancer or immunomodulatory effects in this tumor-bearing mouse [9]. This negative study would be a resource for future study in this area, particularly for Spirulina.

This Special Issue also published two review articles that outlined the benefits of natural products for the treatment of pancreatic cancer and glaucoma. Kim et al. reviewed and analyzed 68 natural products that have anti-pancreatic cancer effects reported during the past five years. They divided this large number of natural products into four categories based on their mechanisms of actions, and presented a summary of their findings [10]. Most of the natural products have been reported to induce apoptosis in pancreatic cancer cells. There are some natural products, including Moringa, Coix seed, etc., that showed multi-functional properties. Overall, this review suggests that some selective natural products can be useful for human pancreatic cancer.

Sim and colleagues provided a comprehensive review of the effects of various dietary supplements in the protection of retinal ganglion cells (RGCs) and in the preservation of the function of the anterior chamber outflow pathways that enable better regulation of intraocular pressure (IOP) [11]. The available data suggest that IOP can be suppressed by natural compounds, including baicalein, forskolin, marijuana, ginsenoside, resveratrol and hesperidin. There are some other plant and plant derived products, including Ginkgo biloba, Lycium barbarum, Diospyros kaki, Tripterygium wilfordii, saffron, curcumin, caffeine, anthocyanin, coenzyme Q10 and vitamins B3 and D that have shown neuroprotective effects on retinal ganglion cells. These data are encouraging but require further extensive investigations in the future to ensure the efficacy and safety of natural products as an alternative therapy for glaucoma.

In conclusion, this Special Issue has highlighted the recent preclinical studies on various natural products with their anti-inflammatory, antioxidative, neuroprotective, cardioprotective, antifibrotic, and anticancer effects, as well as other health benefit effects. Overall, this Special Issue has been a good source of some promising natural products. These include Rubi Fructus, Mokko lactone, YG-1 extract, *D. moldavica* ethanol extract (DMEE), lactoferrin, *S. crispa*, fisetin and quercetin, as well as *S. plagiophyllum* extract. Further clinical studies are needed to validate these encouraging findings.
